# Opportunities for Refinement of Non-Human Primate Vaccine Studies

**DOI:** 10.3390/vaccines9030284

**Published:** 2021-03-19

**Authors:** Mark J. Prescott, Carolyn Clark, William E. Dowling, Amy C. Shurtleff

**Affiliations:** 1 National Centre for the Replacement, Refinement and Reduction of Animals in Research (NC3Rs), London NW1 2BE, UK; 2 Coalition for Epidemic Preparedness Innovations (CEPI), 0473 Oslo, Norway; carolyn.clark@cepi.net (C.C.); william.dowling@cepi.net (W.E.D.); amy.c.shurtleff@cepi.net (A.C.S.)

**Keywords:** 3Rs, animal welfare, biocontainment, biologics, COVID-19, drugs, infectious disease, nonhuman primates, therapeutics, vaccines

## Abstract

Non-human primates (NHPs) are used extensively in the development of vaccines and therapeutics for human disease. High standards in the design, conduct, and reporting of NHP vaccine studies are crucial for maximizing their scientific value and translation, and for making efficient use of precious resources. A key aspect is consideration of the 3Rs principles of replacement, reduction, and refinement. Funders of NHP research are placing increasing emphasis on the 3Rs, helping to ensure such studies are legitimate, ethical, and high-quality. The UK’s National Centre for the 3Rs (NC3Rs) and the Coalition for Epidemic Preparedness Innovations (CEPI) have collaborated on a range of initiatives to support vaccine developers to implement the 3Rs, including hosting an international workshop in 2019. The workshop identified opportunities to refine NHP vaccine studies to minimize harm and improve welfare, which can yield better quality, more reproducible data. Careful animal selection, social housing, extensive environmental enrichment, training for cooperation with husbandry and procedures, provision of supportive care, and implementation of early humane endpoints are features of contemporary good practice that should and can be adopted more widely. The requirement for high-level biocontainment for some pathogens imposes challenges to implementing refinement but these are not insurmountable.

## 1. Introduction

Animal models remain an integral part of the immunogenicity, efficacy, and safety assessment of new vaccines and drugs. They also provide possibilities for research on host–pathogen interactions and the interplay with the host immune system. In some cases, non-human primates (NHPs) provide the best, or even only, models to study these aspects in infectious disease research [1]. Over the last decades, NHP studies have been instrumental in gaining an understanding of the pathogenesis of various infectious diseases and have provided relevant models to develop new therapies (e.g., vaccines against polio, yellow fever, Hepatitis B, and Ebola; identification of the causative agents of infectious diseases such as SARS, typhoid fever, and mumps; and more in-depth understanding of infections such as HIV [2,3,4]). With several emerging viral infections becoming epidemic, NHPs continue to be important animals for investigating human viral diseases [5]. NHPs are highly favored for modeling Nipah, Middle Eastern Respiratory Syndrome (MERS), and Rift Valley fever (RVF), and have been species of choice in the last year for SARS-CoV-2 research and the discovery of vaccines and countermeasures to combat the coronavirus disease 2019 (COVID-19) pandemic [6,7]. Indeed, the global research community has turned to NHP species so heavily for SARS-CoV-2 research that the availability of these animals is scarce [8].

The Coalition for Epidemic Preparedness Innovations (CEPI) is a global partnership between public, private, philanthropic, and civil society organizations. It funds the development of vaccines against deadly diseases such as Nipah, RVF, Lassa fever, and Chikungunya, for which no licensed vaccines are currently available. Of very current interest, two coronaviruses are also in the portfolio of pathogens of importance to CEPI: MERS-CoV, the etiologic agent of MERS, and more recently SARS-CoV-2. The COVID-19 pandemic has brought CEPI to the forefront of not only vaccine development for this novel pathogen, but also in playing a main role in the global initiative COVAX, which aims to guarantee fair and equitable access to novel licensed SARS-CoV-2 vaccines for every country in the world.

Before the pandemic, a clear mandate from CEPI has been the responsible use of NHPs as a valuable research resource, knowing that many of the CEPI priority pathogens must be evaluated in laboratory models of NHP infection because some of these viruses do not infect other laboratory animal species in a way that adequately models human disease. For some pathogens, NHP studies for drug and vaccine development may be considered the pivotal efficacy studies upon which product licensure decisions are made under the US Food and Drug Administration (FDA) “Animal Rule” regulatory approval pathway [9]. Product development studies must be performed under high-quality research conditions with an emphasis on data integrity, and the responsible care and use of the NHP models is integral to performance of successful studies.

Depending on the pathogen’s course of infection and severity of clinical signs, study type (e.g., drug candidate, prophylactic vaccine, therapeutic vaccine) and experimental endpoints chosen, product development studies can potentially result in severe suffering for the NHPs involved, especially untreated or unvaccinated control animals. Optimizing the welfare of NHPs used in such studies is important for CEPI and its funders. Experiments performed without the best welfare approaches risk the collection of poor-quality experimental data, potentially compromising model validity and the ability to detect positive effects of the experimental product under test [10,11,12,13,14]. Sub-standard housing, husbandry, and care practices also result in unacceptable reputational risks to all organizations involved. Public opinion polls show high concern about the use of NHPs in research, and greater approval for in vivo research where steps are taken to reduce animal use and suffering, in line with the 3Rs principles, replacement, reduction, and refinement [4,15].

To help ensure appropriate attention to the 3Rs and high-quality experimental design in funded NHP studies, CEPI relies on the valuable peer review and advice service provided by the UK’s National Centre for the Replacement, Refinement, and Reduction of Animals in Research (NC3Rs) [16]. NC3Rs is a Government-backed organization that works with scientists and institutions across the bioscience sector to discover and implement new technologies and approaches that replace, reduce, and refine the use of animals in research. Under the service, individual research proposals are reviewed by expert staff for opportunities to implement the 3Rs and for compliance with principles in the NC3Rs guidelines “Non-Human Primate Accommodation, Care and Use”, adopted as a condition of funding by CEPI and other funders [17]. The guidelines reflect contemporary good practice and are aligned with housing and husbandry standards provided for NHPs in the European Union (EU). NC3Rs’ feedback is used during funding decisions and when drafting the terms and conditions of grant awards. The Centre is in the privileged position of being able to compare research facilities and practices internationally, and this process has identified opportunities to refine NHP vaccine development studies submitted for CEPI funding and to improve animal welfare without compromising the science.

The NC3Rs has itself funded several research projects aimed at advancing the 3Rs in vaccine testing with NHPs, including awards for the development of an immunologically-competent in vitro model of the human liver to replace NHPs in the assessment of yellow fever vaccine attenuation, and for the transfer between laboratories of an in vitro functional assay to refine efficacy testing of tuberculosis (TB) vaccine candidates by avoiding the need for in vivo challenge [18,19]. The Centre has also worked with the international pharmaceutical and biotechnology industry to embed the 3Rs in the development of monoclonal antibodies (mAbs) as therapies for disease. Non-clinical testing of mAbs poses challenges because their high degree of target specificity can mean that there is either no relevant species to use or the NHP is the only option. By acting as an honest broker for cross-sector data sharing, and analyzing data on over 100 biologics from 15 companies, the Centre has identified opportunities to halve the number of non-human primates used in a typical mAb development program from 144 to 64, whilst supporting patient safety [20,21]. This collaborative work has changed company practice and influenced the addendum to the ICH S6 guidelines on the nonclinical safety evaluation of biotechnology-derived pharmaceuticals [22]. Also of relevance to this special issue, the NC3Rs has been tasked by the World Health Organization (WHO), and funded by the Bill and Melinda Gates Foundation, to carry out an independent and comprehensive review of WHO guidelines for biologics. The international expert working group is evaluating which animal tests are recommended for the batch release and quality control testing of biologics, including vaccines, and what opportunities exist for better implementation of 3Rs principles and alternative test methods, generating recommendations to WHO on how this could be best achieved [23].

CEPI, along with the Bill and Melinda Gates Foundation and US Government agencies such as Biomedical Advanced Research and Development Authority (BARDA) and National Institute of Allergy and Infectious Diseases (NIAID), are funding vaccine development programs for SARS-CoV-2 and other pathogens, yet also increasingly leading the world in discussions of how vaccine development impacts availability of research resources such as NHPs, and the responsibility to use these resources ethically and conservatively. As a way for CEPI-funded laboratories and vaccine developers to speak directly about how to refine their NHP vaccine development studies, CEPI and the NC3Rs organized a joint, non-public workshop in June 2019, hosted by the University of Texas Medical Branch (UTMB), Galveston, TX, USA [24]. The objectives of the workshop were to:

- explore opportunities for refinement of CEPI-funded NHP vaccine development studies, in order to optimize animal welfare and scientific outcomes;
- better align CEPI-funded studies with the NC3Rs NHP guidelines and deliver on public commitments to the 3Rs;
- share relevant data and experience from international laboratories;
- and provide a platform for follow-up work on important concepts such as refinement and standardization of humane endpoints.

Here we share key findings and best practice, and summarize the full recommendations from the workshop to help support other funders, regulators, researchers and laboratory staff to refine NHP vaccine studies and facilitate better animal wellbeing, research quality, data integrity, and public support.

## 2. Refinement Opportunities

### 2.1. Social Housing and Socialization

It is well established that social housing is crucial for the welfare of NHPs, including macaques, marmosets, and other species used in bioscience research [25,26,27]. There is a large body of literature on the negative impact of individual housing on the health and psychological well-being of these highly social animals [28,29,30]. Accordingly, regulations and guidelines mandate or encourage social housing, and this is the default housing configuration at most NHP facilities [17,31,32,33]. A recent international survey of behavioral management practices found that the proportion of facilities housing all of their NHPs socially (i.e., kept with one or more compatible conspecifics in the same cage or enclosure) was 83% in the UK (*n* = 6 facilities), 46% in the EU (*n* = 11), and 32% in the US (*n* = 25) [34].

While exemptions to social housing can be granted for justifiable reasons by the local oversight body (e.g., Institutional Animal Care and Use Committee—IACUC), there is variation in what is considered reasonable grounds for exemption in infectious disease studies, which can last several months. In the aforementioned survey, across all facility types, concerns about cross-infection between animals, monitoring of clinical signs, and anticipated rapid clinical decline of animals were reported as factors preventing social housing by 50%, 29%, and 40% of EU facilities and 75%, 65% and 53% of US facilities respectively, but were not considered constraints by UK facilities. Research facilities in the UK and EU, generally socially house their NHPs throughout natural history, pathogenesis, and therapeutic intervention studies, including during the phase when animals are challenged with an infectious agent (i.e., when under high containment), and this was reflected by UK and French laboratories at the workshop [35,36,37,38]. Perceived problems such as aggression during clinical disease manifestations, or healthy animals injuring sick companions, have not been observed. Similarly, the National Microbiology Laboratory of the Public Health Agency of Canada (PHAC), which at the encouragement of its IACUC has pair-housed macaques in BSL-4 for over seven years, also reported that with familiarization and acclimation processes, the pair-housed animals are not aggressive towards each other. When reporting study findings, researchers should give details of the social configurations used (e.g., pair housing), the timings of these if changed through different phases of the study, and the reasons for any individual housing.

In the USA, research with non-human primates is regulated by the Animal Welfare Act and its regulations, which are administered by the Animal and Plant Health Inspection Service (APHIS) of the US Department of Agriculture (USDA). Separately, regulations for handling pathogens are set by the Division of Select Agents and Toxins (DSAT) of the Centers for Disease Control and Prevention (CDC). Most facilities tend to separate NHPs before being moved into rooms for infectious disease studies or before the challenge phase [39]. Practicality, staff safety, and scientific concerns (around animal cross-contaminations and accurately tracking and managing animal biosamples that potentially contain infectious material/select agents) are also identified as barriers to social housing. However, it is unclear why this should be the case in some world regions and not in others. It is notable that in the UK, vaccine efficacy studies conducted with pair- or group-housed NHPs have been accepted by the FDA, and the facilities have been inspected by CDC personnel to select agent criteria, with no issues raised regarding social housing.

The consensus is that for reasons of both good animal welfare and good science, NHPs should be socially-housed during vaccine studies, and with appropriate infrastructure and expertise this can be achieved in most cases, especially during the phase of the study where animals are held vaccinated but prior to the challenge phase. Long-standing experience shared at the workshop by PHAC, Public Health England (PHE), the Defence Science and Technology Laboratory (DSTL), and the French National Institute of Health and Medical Research (INSERM) demonstrates that social housing is also possible during the challenge phase. In the opinion of these research teams, pair- and group-housed animals are more content, calmer, and may recover more quickly from procedures than singly housed animals on studies involving high containment pathogens. Social buffering studies (including in BSL-2) have shown the powerful role of social housing in mitigating reactions of NHPs to stressful events, enhancing immune responses, and optimizing the ability to cope with disease, potentially leading to models that are more representative of the human condition [13,40,41,42,43,44,45]. It is conceivable that working with calmer, less stressed subjects could also result in less variable data and more reproducible findings, which may enable a reduction in NHP use, though to our knowledge, this has not yet been proven in specifically designed studies. It is incumbent upon organizations like CEPI and the NC3Rs to fund such studies to validate the scientific benefits of social housing.

There is a large literature on how to set up and maintain compatible pairs/groups of NHPs [13,46]. Breeding on-site, or else a strong relationship with external suppliers who can provide biographical information and behavioral compatibility data, enables the identification of compatible animals for experimental use, minimizing the likelihood of aggression occurring later [47]. These practices also facilitate training of the animals in preparation for studies. Such animals may come at higher prices, but obtaining pre-established pairs or groups takes the burden off researchers and care staff at the experimental facility, reducing the investment of time and effort. In addition, cage groups can be organized such that animals are familiar with their nearest neighbors before being moved into the high containment area.

Laboratories that group house up to six animals per group report no issues in terms of results being confounded by cross-infection provided there is careful allocation of treatments (i.e., all animals housed in each group receive the same treatment, such as the infectious dose and/or vaccination regimen) and refinement of challenge doses. Blinded studies sometimes require mixing of experimental treatment groups, or at least coded cage-labeling, which is operationally more difficult, but possible. One solution could be personnel-based: it may be possible to have two technical teams, one which is aware of animal group assignments, for the purpose of administering vaccines or therapeutics, and a second blinded team unaware of group assignments to perform the daily clinical health assessments, especially if observations which can be subjective are a primary endpoint for the study. Obviously, socially housed individuals need to be well-identified (e.g., via tattoo). Where surgical procedures are necessary, compatible pairs/group can be temporarily separated for post-surgical recovery and wound healing. Some experiments will be designed to observe potential disease recrudescence; for example, after completion of an experimental treatment, animals may be held for an extra month to determine if latent infection might reactivate. Pair housing might confound this situation because the second animal may be re-exposed to the infection, and could raise questions about the efficacy of the experimental vaccine or immune status post-challenge. This situation would require careful observation, immune assessments, and evaluation of both pair-housed animals before the study were brought to a close.

Monitoring several animals in a social group simultaneously is time efficient for the researcher and care staff. In addition, behavioral signs of illness (e.g., changes in locomotion, balance, coordination, and peer interaction) are often easier to detect in socially housed NHPs because they express a wider range of natural behaviors than do singly-housed animals. Any deterioration in well-being shows a marked contrast against both the behaviors of cage mates and the previous behaviors of the affected animals. Measurement of individual food intake and excreta is a challenge in social groups, but these parameters may not be primary endpoints in vaccine or therapeutic efficacy studies, but rather toxicology studies not involving an infectious agent. If food and water intake are important endpoints, then planning for paired housing and quantitative intake assessment methods should be carefully considered in the study design. Intake can be observed on a pair/cage basis instead and used along with other indicators of well-being as part of a holistic approach, such as body weight measurement, noting of feeding habits during feeding times, behavioral observations via closed-circuit television (CCTV) by experienced staff (i.e., familiar with the individual animals and their “normal” behavior), and clinical chemistry and virology following blood sampling.

### 2.2. Enclosures and Enviromental Enrichment

For good health and psychological wellbeing, captive NHPs require a complex and stimulating environment that provides them with opportunity for social interactions, exercise, and the ability to express a wide range of behaviors appropriate to the species [46,48,49]. Unfortunately, this is not always provided to NHPs housed under high biocontainment. Where this is the case, pre-challenge work, such as the vaccination phase of a study, should be done in the normal colony environment or high-quality standard accommodation area, because this approach can act as a refinement by minimizing the amount of time spent in relatively small, unenriched cage units of the high containment environment.

In the experience of EU NHP facilities, high containment studies can be run in the group-housed condition in relatively large and well-enriched pen-style enclosures (i.e., 3.6 m^3^/127 ft^3^ for two macaques >3 years of age, which is the legal minimum in the EU under Directive 2010/63/EU [31]) yet still be compliant with safety and good laboratory practice (GLP) standards. Structural enrichment (e.g., ropes/rope ladders), toys/manipulanda (e.g., balls, wooden dumbbells, mirrors), and foraging devices (e.g., puzzle feeders, cardboard forage boxes, forage trays with floor substrate such as Enviro-dri^®^ or SizzleNest^®^) are commonly provided, all of which are easily decontaminated or disposed of at the end of studies. A high level of environmental enrichment increases the scope for scoring relevant clinical signs because NHPs often change the level of interaction with enrichment items when they experience adverse effects, and this may occur before other detectable clinical signs are present.

Cages used for studies involving infectious agents need to be able to withstand robust decontamination processes, such as autoclaving and fumigation. Whilst stainless steel is the obvious choice as a material for such cages, other materials can be used to provide a softer and less noisy environment, such as Trespa^®^ for cage partitions and polypropylene for platforms, shelves, and perches.

**Figure 1:**
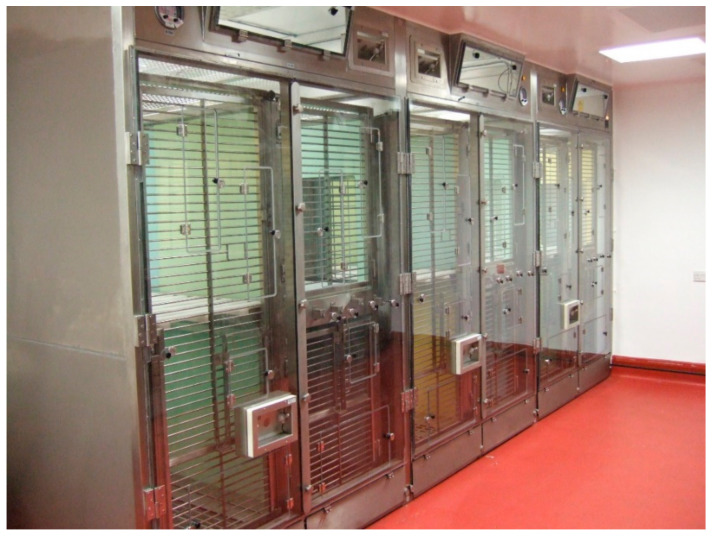
Containment system for group housing of macaques at Public Health England (PHE). Three cages are shown, each consisting of four units. (Image: Mike Dennis, PHE).

PHE has developed a state-of-the-art containment system which balances the need for staff safety at all stages of the experimental process, whilst addressing the requirements of macaques for space, environmental enrichment, and social interaction (Figure 1). The system has been used to study a variety of infectious agents, including SARS-COV-2. It allows group housing whilst offering quantifiable operator protection during infection, experimental sampling, and husbandry. The containment system utilizes the principle of directional airflow away from the operator towards the rear of the cage in order to protect staff (staff still wear full personal protective equipment [PPE]). With correct operation, this flow is maintained at a minimum of 0.7 M/second and the air extracted by a total-loss room air handling system via high efficiency particulate air (HEPA) filtration. A series of interconnected cages are placed inside modular booths that have a clear rigid plastic screen at the front of the system. This screen allows good observation of the caged macaques whilst providing protection from physical contamination by urination, defecation, cough, throwing of objects, or by reaching through from the cage. Each screen is fitted with flap valves that control the velocity of the air into the system and each has a number of small access doors placed in strategic alignment with the cage front to allow animal care staff access to replace food and water, change bedding, and sedate the occupants for subsequent procedures. Further operator protection is provided by using a transfer box for sedated animals and a downdraught table for more risky procedures, such as blood sampling, bronchioalveolar lavage, X-rays, close clinical assessment, and necropsy. Additional details are given under “Housing” on the NC3Rs Macaque website [50].

DSTL has also developed bespoke caging for its BSL-3/4 studies with vasectomized male-female pairs of marmosets. Cages are constructed of stainless steel, with a polypropylene section at the rear to ensure full telemetry coverage, and housed in a rigid half-suit isolator maintained at negative pressure [51]. The animals are provided with a good amount of cage furniture (e.g., removable nest box, veranda, and wooden/plastic perches), treats, and a varied diet in high containment. A hammock positioned at the front of the cage facilitates observations of the animals, even by CCTV and at night. A cassette of nylon netting can be inserted at the top of the cage and wound down to bring the animal to the base of the enclosure quietly and gently, which enables safe restraint and handling, avoiding the need for hand capture or use of a heavy cage squeeze-back mechanism [52].

Where facility architecture (e.g., size of rooms, dimensions of doors, size and configuration of autoclaves) prevents the purchase of more spacious pen-style enclosures, existing caging systems can be customized to meet the animals’ needs for companionship and provide greater space without decreasing housing capacity or incurring major financial cost; for example, side panels can be removed to link and combine standard one-over-one caging units. Pair housing need not decrease animal capacity since the pair can occupy the combined space previously given to the two animals separately. The squeeze-back mechanism can be retained in one of the units, so that animals can still be restrained, enabling greater cage enrichment in the other/adjacent unit. Animals can be readily trained before the study to shift on command into the unit with the squeeze-back and will reliably do so. In this way, there is no greater risk to staff safety and no need for new caging or larger squeeze-backs, which would be unsafe and unwieldy in biocontainment.

### 2.3. Animal Training, Sedation, and Selection

When working with dangerous pathogens, physically restraining NHPs to administer substances is not a safe option, and where significant volumes of blood are required it would be difficult to sample safely from an unsedated individual. Chemical restraint is therefore widely used, but researchers need to be aware of the impact of sedation on physiological processes and translational relevance to clinical settings. For instance, ketamine sedation was recently found to blunt cytokine levels in rhesus and cynomolgus macaques compared to awake cooperating animals [53]. It is associated with a prolonged reduction in daily food intake in NHPs [54], which can lead to significant body weight loss when used repeatedly. Where sedation is used, fully and quickly reversible anesthetic agents are preferable since these enable rapid recovery (e.g., eating and drinking) within minutes and swift return to the social group [55].

Regardless of whether sedation is used, NHPs should be trained to cooperate with study procedures and adapt to the high containment environment. Effort put into training and acclimation will be recouped later in facilitating the performance of the study and animal monitoring and welfare, leading to higher quality, less variable data [40,56,57,58,59]. NHP training can be conducted at the breeding unit, in the pre-study phase, and/or before transfer to containment housing or high level containment, and should be performed consistently amongst researchers and technicians. Training approaches could be considered important methods that impact the results of the study; therefore, training methods should be standardized through the use of standard operating procedures (SOPs), staff training sessions, and CCTV monitoring to deliver consistency in technique. There are some excellent resources on humane training methods available in the literature [60,61,62].

Good practice identified at the workshop includes:

- Training animals using positive reinforcement (e.g., food rewards) to station (approach a specific location) or target (touch a specific object) on command, including when in pairs/groups, so that there is reliable voluntary control of animal movement when housed in the containment environment and all individuals can be dosed without interference [63,64].
- Training to come forward and present themselves for intramuscular injection of a sedative, so that procedures such as X-ray, weighing and temperature measurement, which are vital to clinical assessment, can be conducted safely and with minimal stress caused to the animals. Marmosets have also been trained within 7–10 days to enter a removable capture box which can then be connected to a gaseous anesthesia unit in a biosafety cabinet for sedation without handling the animal [65].
- Training to sit on a scale or in a weighing bucket (for small monkeys, such as marmosets), so that staff members do not need to handle the animals for weighing once they are on-study (resulting in less stress for animals and staff) [66].
- Training to take liquid (e.g., fruit juice, milkshake) from a syringe, which can later be used to administer potentially bitter tasting therapy or medication as required, rather than requiring anesthesia to administer dose [64].
- Assisting adaptation to the “new” containment environment by transfer of familiar items (e.g., scent-marked nest box of marmosets).
- Interacting with the animals pre-study to familiarize them with the personnel involved, the appearance of staff in full PPE, and human behavior. This also enables staff to get to know individual animals and how each behaves, meaning they can then more easily identify changes from normal, facilitating humane endpoints based on changes in behavior.
- Integrating animal training into the experimental protocol.

PHE has cynomolgus macaques (two origins) and rhesus macaque (Indian) breeding colonies on site, which means they can select the best species/model for the disease. They have published on species/strain differences in model development [67], and it is worth considering what implications colony origin and disease differences may have on model refinement and translation [68,69]. Use of animals from different populations or vendors may result in wide variation in endpoints; an example of this was given at the workshop. In general, it is desirable to use animals from one source on a given study or series of studies, to support collection of consistent data.

### 2.4. Humane Endpoints

A key component of refining NHP vaccine studies is the identification and implementation of humane endpoints. These can be defined as the earliest reliable indicator in an animal of pain, distress, suffering, or impending death on the basis of which the animal is euthanized, treated, or removed from the study [70]. By substituting these planned triggers for intervention in place of more severe experimental outcomes, such as advanced pathology or death, humane endpoints prevent or alleviate unnecessary pain and distress, whilst still meeting the desired experimental objectives.

Participants at the workshop agreed that it is essential to identify accurate humane endpoints prior to study initiation, and to monitor animals at an appropriate time and frequency, to enable the earliest possible euthanasia decision and avoid moribundity and spontaneous animal death in severe studies. The humane endpoint guidelines of the US Association of Primate Veterinarians ([71], p.7) state: “A moribund condition indicates an animal is in a severely debilitated state and in terminal distress. Moribund condition and death should be avoided as study endpoints (unless there are no alternatives) and must be scientifically justified and approved by the IACUC. Unless scientifically justified and approved by the IACUC, all moribund NHPs should be immediately evaluated by a veterinarian and euthanized.”

Aside from the animal welfare benefits, identification of early endpoints can improve scientific results by helping to ensure that biologic samples are minimally degraded and of limited variability due to adverse effects of severe illness (e.g., inability to obtain food or water, dehydration, hypothermia) [72]. Using early endpoints also minimizes the influence of adverse effects unrelated to the infection on disease development and animal death, which would otherwise complicate determination of an organism’s pathogenicity [73].

A comprehensive review of humane endpoints used in NHP vaccines studies is beyond the scope of this article. Typical endpoints for disease models of infection with CEPI-prioritized pathogens include, but are not limited to, body temperature, inappetence, weight loss, slowness or lack of responsiveness, and/or lying prostrate. Different viral models, and even different strains of the same virus, may require different endpoints due to varying aspects of unique pathogenesis caused by those viruses/strains. The goal of the study/vaccine, inoculation route, and pathogen dose level may also influence the choice of endpoints. For example, if the goal of a vaccine is to prevent all signs of disease, then selection of early endpoints, such as a change in body temperature, could suffice for drawing the study conclusions; whereas late onset therapeutic intervention would necessitate significant and more advanced clinical signs.

Humane endpoints should be continually refined over time as more data become available from studies using the specific model. Compiling and reviewing historical datasets can identify trends, enabling further or earlier endpoints to be developed. Participants at the workshop felt the field needs more natural history studies of CEPI pathogens for the specific purposes of defining the exact disease progression in the models and facilitating endpoint identification and validation. Greater data sharing and collaboration between research and veterinary staff could also assist the development and refinement of humane endpoints. It would help to standardize endpoint criteria across institutions so that similar study outcomes could be observed. For endpoints to be used consistently across laboratory sites, thorough training of staff members is required. All personnel performing endpoint criteria assessments need to be able to recognize the signs of disease for the infection models and apply consistent scores to clinical signs of illness and changes in behavior. Veterinarians need to also take a consistent approach to observations and treatment, when required. Structured welfare assessment/humane endpoint score sheets are valuable tools that can be used to monitor and document behavioral and physiologic parameters that are predictive of changes in clinical conditions [74]. These should be available to all relevant staff and preferably be included in training on standard operating procedures (SOPs). One group that worked to harmonize endpoint criteria across multiple institutions was the Filovirus Animal Non-clinical Group (FANG), a US interagency group focused on facilitating the development of filovirus medical countermeasures [75]. Full adoption of a standardized scoring system was not achieved but there was greater harmonization of endpoints across laboratories [76]. Discussions regarding changes in established endpoints need to include not only the researchers but also veterinary staff and IACUC members.

Clinical scores are sometimes accompanied by secondary endpoint measurements, if available, such as hematology and serum chemistry parameters measured on laboratory instruments [77]. At this time, reliable secondary blood measurement endpoints are not uniformly described for Lassa, Nipah, MERS-CoV, or other CEPI pathogens. Better datasets, such as those that could be collected through natural history studies, would help inform changes in blood chemistry parameters that may be predictive of lethal disease and/or serve as early biomarkers for euthanasia decisions. Such an approach was employed for assessment of the Ebola virus rhesus macaque model, which led to several institutions adopting secondary endpoints of temperature change and specific clinical chemistry values [78,79].

When endpoints are measured during a study, baseline (pre-pathogen exposure) measurements should be taken for each animal and changes relative to that animal’s baseline should inform decisions about clinical scoring. Animals should be assessed at times and frequencies that will best help to identify the early onset of harmful effects. Similarly, experiments should be scheduled so that an adequate number of trained research personnel are available to assess animals when they are most severely affected and require intensive monitoring [72]. For marmoset infectious disease studies at DSTL, welfare monitoring is performed at 8-h intervals, including CCTV and physical observation, and there is 24-h coverage by experienced scientists and technicians. Once animals begin to show clinical signs, observation is increased to every 4 h and then moves up to continuous monitoring until the predetermined humane endpoint criterion is reached.

Telemetry is an innovation that is not as widely used as it could be for implementing humane endpoints in NHP infectious disease studies, perhaps due to the cost for installation in high containment laboratories. Telemetry offers a large, accurate, and objective dataset that can be used to refine endpoints such as body temperature, blood pressure, ECG and heart rate, respiratory rate, activity levels, and even glucose in some implant models. Pre-implanted animals can be ordered if implantation surgery is not possible at the receiving BSL-4 laboratories. If more facilities used telemetry, then model development could perhaps be better standardized across facilities through use of the same endpoint criteria (e.g., temperature, hypotension).

At DSTL, telemetry devices are implanted 4 weeks prior to challenge to enable monitoring of the core body temperature of individual marmosets in real time. The facility can record from 16 pair-housed animals at once. Marmosets exposed to *Burkholderia pseudomallei* show clinical signs 12–24 h after temperature increase. Once there is observation of a sharp temperature decline following the febrile response, the animals are euthanized before more severe disease signs are observed [35]. A decline to 39 °C has been defined as the humane endpoint criterion (in combination with clinical signs), as the disease model demonstrates little variation between animals. Marmosets at this facility also wear an activity tracking device (Actiwatch) on a collar, from which data can be downloaded at the end of the study [80]. The animals become less active as disease progresses, so this change in behavior may be used as an endpoint in some models, together with other data. The CCTV camera at the top of the cage, which enables monitoring with minimum disturbance, is particularly useful at the end stage of disease, when external stimuli such as entrance of a caretaker into the room may cause the animal to react and hide its illness/vulnerability [51]. The laboratory is currently investigating a Data Sciences International (DSI) telemetry implant that enables measurement of core body temperature, EEG/ECG, HR, BP, and activity, and can synchronize with camera data.

Telemetry has also been used extensively in the establishment of a model which has been submitted under the FDA Animal Model Qualification program. The cynomolgus macaque model of inhalational tularemia for assessment of therapeutics was developed at multiple institutions under a NIAID program. As with the marmosets described above, telemetry devices were implanted in the animals to allow monitoring of core body temperature. In this model, a fever early in the disease course was used as a trigger for antibiotic treatment. Late in the disease course, hypothermia was consistently observed and was incorporated into euthanasia criteria [81].

Medical imaging (such as CT and PET scanning) is another useful innovation, but it is less widely available than telemetry, especially in biocontainment facilities. For its aerosol challenge studies with macaques (e.g., TB), PHE hires portable clinical scanners to come on site, and the animals are transferred into a negative pressure imaging pod. This allows them to monitor disease progression and look for disease effects (lesions and nodal enlargement), instead of leaving animals to suffer severe disease, euthanizing at an anticipated timepoint, and doing histopathology. For tuberculosis studies, CT scanning also allows evaluation of the challenge dose received by counting primary tuberculin lesions, and this has in turn enabled the use of much lower, clinically realistic challenge doses that can be used to evaluate efficacy whilst improving welfare through reduced disease burden. PET-CT methods represent a longer process for imaging capture, so the laboratory uses high frequency jet ventilation (HFJV) to stop imaging motion artefacts caused by breathing [82]. It has published a methodology for disease evaluation from CT images that aligns with clinical scores seen at necropsy, allowing much earlier detection of treatment effects and hence shorter time courses for studies in high containment [37]. CT and PET/CT is also being used by multiple facilities for analysis of COVID-19 disease in macaques which allows assessment of disease onset and timing of treatment interventions [83,84,85].

### 2.5. Supportive Care

Supportive care, such as fluid replacement and pain relief, is another means of refinement. Participants at the workshop considered that provision of supportive care is important for humane reasons and expected by ethics committees (e.g., IACUCs). Vaccinated animals need to be healthy upon entry into the challenge phase of the study, so animals displaying idiopathic dehydration or diarrhea during the vaccine phase are generally placed under watch of a veterinarian, and given fluids and common medications for relief of symptoms. Some veterinary treatments, such as immunosuppressants, may be contraindicated in the challenge phase, though palliative care should always be possible and does not seem to affect disease outcomes in the experimental model (e.g., offering soft or moistened food; additional, hydrating fresh fruit/vegetables; ice cubes; oral rehydration solution; liquid via a syringe; or moving the water bottle closer to an animal showing muscle weakness).

The type of supportive care offered should be selected carefully since treatments may interfere with the humane endpoint criterion, correlate of protection or biomarker that is being measured. So, for example, where the febrile response is the primary indicator of disease and essential for the humane endpoint, fever reducing medications or non-steroidal anti-inflammatory drugs (NSAIDs) may not be appropriate. The plan for supportive care should be detailed prospectively in the study design and agreed upon among the stakeholders (e.g., product development sponsors, regulatory agencies) at the outset. Considerations such as whether each animal will receive care when meeting a trigger point, whether every animal will receive the same supportive care uniformly, and whether staff are suitably trained and competent to detect key signs of illness, should be discussed during protocol design. Some respondents at the workshop commented that administration of supportive care might increase the risk to staff (e.g., if it involves handling of sharps, or extra hours spent in high containment), so the biosafety considerations need to be considered alongside animal welfare.

Where the effect on correlates of infection or protection is unknown, it may be necessary to test each intervention to determine its effect on the model. Participants had experience with administering subcutaneous or intravenous fluid therapy to models of Lassa or Ebola virus infection in macaques. In one specific case, supportive care did not offer a statistically significant survival benefit or extend mean time to death, which means that adopting these practices during experimental studies of a particular therapeutic’s efficacy should not confound the study results and may importantly afford the animal some alleviation of pain or suffering [86]. This group also tested whether Tylenol (Acetaminophen), as a pain reliever that is processed by the liver, had any effects on liver enzyme levels during Ebola virus infection of rhesus macaques, which it did not (even though Ebola virus is known to cause virus-induced liver cytopathicity, which might potentiate further damage after Tylenol administration). Other drugs may potentiate liver damage in virally infected animals, so a plan for testing is important.

The use of suitable analgesics should be considered when it is expected and predicted that the challenge process could be painful at any stage [87,88]. If analgesia is used, the type of analgesic, dose, and treatment regime need to be empirically examined to assess not only whether it is effective and beneficial, but also to confirm that it does not materially interfere with the course of the disease or identification of humane endpoints [89].

## 3. Conclusions

There is considerable scope to further refine NHP vaccine studies to minimize harm to the animals involved and to maximize data quality, in line with societal, regulatory, and funder expectations. Researchers, veterinarians, technical and biosafety staff, regulators, and funders should therefore work together to improve existing practices and challenge dogma. With sufficient will, knowledge, training, and resources, NHP vaccine studies can be conducted to GLP, or other systems that assure data quality and integrity, while meeting genuine high standards of animal welfare. For example, experience has shown pair- or group-housing of NHPs does not have a negative effect on the science or the ability to do experiments in BSL-4 conditions, and biosafety departments can adequately quantify the methods used for keeping staff safe. Safety is, of course, a prime consideration at high biocontainment levels and training of staff to ensure consistent use of de-risked practices is essential [90]. Institutional SOPs should be written and reviewed for safety and risk management around all practices, including social housing.

Funders of NHP research are placing increasing emphasis on the 3Rs and are important drivers for improving standards, helping to ensure such studies are legitimate, ethical, and high-quality. Discussion at the workshop led CEPI in August of 2019 to release a call for proposals “High Containment Studies to Support Product Development” with the goal of setting up contracts with preferred laboratories that meet standards laid down by the NC3Rs and high regulatory quality standards, such as GLP. As part of this, CEPI agreed to make investments in infrastructure and training to help laboratories achieve the required standards. Ultimately contracts were signed with several facilities in the EU for animal studies at BSL-3/4, where novel animal telemetry, plethysmography, and activity-monitoring equipment were purchased, to enrich the animals’ housing as well as expand the data endpoints collected at these laboratories. In the USA, a CEPI contract to the University of Pittsburgh facilitated an upgrade to its NHP caging, which enabled social housing in an expanded-size enclosure that approaches EU caging standards. These investments, made at the early stages of the pandemic, have already contributed to achievements in performance of 3Rs compliant studies supporting COVID-19 vaccine development. In parallel, the NC3Rs has established a working group with the US National Toxicology Program Interagency Center for the Evaluation of Alternative Toxicological Methods (NICEATM) and other agencies to coordinate global efforts to use microphysiological systems (organ-on-a-chip and other complex multicellular in vitro models) to reduce reliance on in vivo studies of COVID-19 and future infectious diseases.

Given their importance for both animal welfare and study quality, refinement measures employed in NHP vaccine studies should be properly reported in published manuscripts, in accordance with the ARRIVE guidelines [91]. For further guidance and resources on improving NHP care and use, including dedicated websites, an annual symposium, and data sharing working groups, visit the NC3Rs website (www.nc3rs.org.uk/nhpwelfare; accessed on 18 March 2021).
